# Desire for childbearing in the view of Iranian parents: A qualitative study

**DOI:** 10.1371/journal.pone.0330658

**Published:** 2025-08-22

**Authors:** Maryam Mesbah, Sulmaz Ghahramani, Reza Amani, Elham Rahmanipour, Mohammad Ghorbani, Ahmad Kalateh Sadati, Sara Sadeghieh, Kamran Bagheri Lankarani

**Affiliations:** 1 Health Policy Research Center, Institute of Health, Shiraz University of Medical Sciences, Shiraz, Iran; 2 School of Pharmacy, Shahid Beheshti University of Medical Sciences, Tehran, Iran; 3 Department of Veterinary Medicine, Babol Branch, Islamic Azad University, Babol, Iran; 4 Immunology Research Center, Mashhad University of Medical Sciences, Mashhad, Iran; 5 Orthopedic Research Center, Mashhad University of Medical Sciences, Mashhad, Iran; 6 Department of Social Sciences, Yazd University, Yazd, Iran; Addis Ababa University, ETHIOPIA

## Abstract

**Background and Aims:**

Like many other countries, Iran is facing a declining population growth rate. While previous research has primarily focused on childless individuals or parents of only one child, limited attention has been paid to families who, despite already having two or more children, continue to desire larger families. This study examines childbearing motivations among Iranian parents with two or more planned children.

**Methods:**

This explorative qualitative study was conducted in 2023 across three provinces in Iran. A total of 46 participants were purposively selected. Data were collected through semi-structured interviews, and thematic analysis was used to identify core themes related to childbearing motivations.

**Results:**

Participants’ desire to have more children was shaped by six major themes: family altruism, value–attitude motivation, real happiness, resilience development, institutional support, and multifaceted balance. These themes reflect a dynamic interplay of emotional, cultural, and practical considerations in shaping fertility intentions.

**Conclusion:**

The findings indicate that both personal and societal factors contribute to parents’ motivation for having more children. Institutional support also emerged as an important influence in the decision to pursue larger families. Understanding these motivations can inform policies aimed at encouraging childbearing among younger generations and help guide the development of integrated strategies and educational initiatives. Future quantitative research is recommended to assess the relative importance of each identified theme.

## Introduction

Understanding global population dynamics is essential for achieving the Sustainable Development Goals (SDGs), particularly SDG 3 (Good Health and Well-Being) and SDG 5 (Gender Equality), as these are closely linked to fertility trends, maternal health, and evolving gender roles in parenting [1]. Globally, the annual population growth rate has declined significantly—from 2.1% in the late 1960s to 0.8% in 2021 [2]. Similarly, Iran has experienced a dramatic decrease, with population growth falling from 5.3% in 1982 to 0.8% in 2021, primarily due to reduced fertility rates [3].

Replacement-level fertility—approximately 2.1 children per woman—is needed to maintain population stability in the absence of migration and mortality. Persistently low fertility rates below this level carry profound consequences: population aging, higher dependency ratios, reduced labor forces, and increased strain on health, pension, and social support systems [4,5]. These trends are apparent across East Asia; for example, South Korea’s fertility rate hit a historic low of 0.81 in 2021 [6], while Singapore’s total fertility rate (TFR) fell to 1.10 in 2020 and 1.04 in 2022 [7,8]. Japan’s TFR also dropped from 2.06 in 1970 to 1.29 in 2020 [6]. These patterns highlight the urgency of integrating fertility concerns into long-term planning for economic development, innovation, and sustainability.

In Iran, fertility has become a policy focus amid growing concern about demographic shifts and an aging population. Studies have documented fertility decline across all provinces and social strata, revealing increasing convergence in reproductive behavior nationwide [9]. Notably, sub-replacement fertility has become widespread, with some regions experiencing extremely low birth rates [10].

To inform policymaking at both public and Ministry of Health levels, it is crucial to understand the factors that influence reproductive behavior in Iran. Identifying the motivations of parents who have planned to expand their families offers an important counter-narrative to prevailing low-fertility trends. While most prior studies have focused on retrospective justifications among nulliparous individuals or parents with only one child, this study centers on those who have actively planned to have more children. This distinct perspective highlights proactive motivations for higher fertility and provides a more nuanced understanding of population dynamics in a low-fertility context.

Qualitative research provides the necessary depth to examine participants’ subjective perceptions, values, and intentions, capturing the complex array of beliefs and meanings associated with fertility decisions [11]. While existing literature is largely quantitative and often centered on single or childless individuals with limited interest in marriage or family life [12–14], some studies have explored voluntary childlessness or single-child decisions in various cultural settings, driven by personal, social, or economic factors [15–18]. Other investigations have examined how psychological, relational, and policy-related constraints shape fertility behavior in low-fertility societies [19–22].

However, few studies have focused specifically on Iranian parents who already have children and still desire more. To fill this gap, this study asks: What factors influence childbearing decisions among parents with two or more planned children? The research explores the motivations and perspectives of these parents across three provinces in Iran.

## Methods

### Study design

This explorative qualitative study was conducted using a thematic analysis approach between November 2022 and July 2023 in Iran. The research involved 46 participants across three provinces: 16 from Rasht (Gilan), 13 from Shiraz (Fars), and 17 from Mashhad (Khorasan Razavi). These cities were selected to capture Iran’s geographic, ethnic, economic, and cultural diversity, reflecting varied fertility contexts—low (Rasht), medium (Shiraz), and high (Mashhad) TFR.

Rasht, located in northern Iran, was among the first areas to experience sub-replacement fertility, shaped by early industrialization, widespread female education, and delayed marriage [23,24]. Shiraz represents a demographic transition zone characterized by moderate fertility, a mixture of traditional and modern norms, economic heterogeneity, and relatively high female education levels [25]. Mashhad, a major eastern metropolis and religious center, maintains higher fertility and stronger traditional family norms, providing a contrasting socio-demographic landscape and enhancing the transferability of findings [26].

Participants were stratified by gender and represented a range of socio-economic backgrounds. Economic status was assessed based on self-reported household income relative to expenditure, a culturally appropriate method in the Iranian context where direct income reporting may be unreliable [27,28]. Participants were categorized into: (1) income greater than expenditure (high status), (2) income equal to expenditure (middle status), and (3) income less than expenditure (low status). Eligible participants included women aged 15–49 with three or more children, those currently pregnant with a third child, or those planning or expressing a desire to have a third child, including cases where twins or triplets resulted in a total of three children. Their husbands were also included if they met the criteria. Individuals without informed consent were excluded. Recruitment continued from November to December 2022 until data saturation was achieved, ensuring thematic and interpretive adequacy. Data collection and preliminary analysis were conducted concurrently from December 2022 to April 2023 in an iterative manner, with final theme refinement and manuscript preparation occurring from May to July 2023. To enhance reflexivity, team discussions and reflective journaling addressed researcher positionality, power dynamics, and potential biases during data collection and analysis [29].

### Sampling and data collection

Data were collected through semi-structured, in-depth interviews conducted in private and culturally appropriate locations to encourage candid responses. Interviews began with general questions such as “What motivated your desire to have children?” and “What factors influenced your decision to plan for more than two children?” Follow-up probing questions were used to explore participants’ responses in greater depth. Each interview lasted approximately 20–40 minutes and was complemented by field notes recorded immediately afterward.

Saturation was monitored iteratively and considered achieved when three consecutive interviews yielded no new codes or themes [30]. Three researchers independently reviewed transcripts and held consensus meetings to ensure consistency and comprehensive interpretation. The final sample size of 46 exceeded the minimum threshold for thematic analysis due to the inclusion of diverse participant profiles across provinces [31].

### Data analysis

Inductive thematic analysis was performed, with codes generated directly from the data. The process followed Braun and Clarke’s (2006) six steps: familiarization, initial coding, theme searching, sub-theme development, thematic refinement, and final report production [32]. Open coding and a constant comparative method were used to refine categories and themes, supported by memo writing and documentation to minimize bias [33–35]. The research team had experience in qualitative research, and the SRQR checklist was used to enhance quality [36].

### Trustworthiness

To ensure rigor, Guba and Lincoln’s (1994) trustworthiness criteria were employed [37]. Peer debriefings supported analytic consistency. Member checking was conducted with selected participants to confirm accuracy of thematic interpretations. Feedback from experts and peers was incorporated, and an audit trail documenting coding revisions was maintained.

### Ethical considerations

Before interviews began, participants received full information about the study’s purpose, their rights, and the reasons for audio recording. Written informed consent was obtained from all participants. Audio recordings were only made with permission; otherwise, notes were taken. Participants were informed of their right to withdraw at any time. Ethical approval was granted by the Research Ethics Committees of Shiraz University of Medical Sciences (ID: IR.SUMS.REC.1401.721).

## Results

This study included a diverse group of participants whose demographic characteristics helped contextualize their motivations for childbearing. Female participants had a mean age of 35.60 ± 7.41 years, while male participants were slightly older, with a mean age of 42.75 ± 6.69 years. All male participants were employed full-time. In contrast, only 23.3% of female participants were employed, with the highest rate in Mashhad (41.7%) and the lowest in Shiraz (10%). Regarding family size, the majority of participants had three children (36.96%), with 17.39% having four or more. This variety ensured that both current and prospective experiences of parenting three or more children were represented. These demographic details are summarized in Table 1.

**Table 1:** The sociodemographic characteristics of the participants with two or more children in three cities of Iran.

		Rasht	Shiraz	Mashhad	Total
**Age Group**	20-29	0 (0.0%)	1(7.69%)	10 (58.82%)	11 (24.44%)
	30-39	5 (31.25%)	5 (38.46%)	6 (35.29%)	16 (35.56%)
	40-49	9 (56.25%)	6 (46.15%)	1 (5.88%)	16 (35.56%)
	50+	2 (12.5%)	1 (7.69%)	0 (0.0%)	2 (4.44%)
**No. of Children**	2	3 (18.75%)	2 (15.38%)	16 (94.12%)	21 (45.65%)
	3	9 (56.25%)	7 (53.85%)	1 (5.88%)	17 (36.96%)
	4+	4 (25.0%)	4 (30.77%)	0 (0.0%)	8 (17.39%)
**Mean Age**	Female	41.90 ± 4.65	37.60 ± 5.56	28.67 ± 4.91	35.60 ± 7.41
	Male	44.33 ± 3.72	45.33 ± 9.61	36.40 ± 4.72	42.75 ± 6.69
**Sex**	Female	8 (50.00%)	10 (76.92%)	12 (70.59%)	30 (65.22%)
	Male	8 (50.00%)	3 (23.08%)	5 (29.41%)	16 (34.78%)
**Economic Status**	Income > expenditure (high)	5 (32.25%)	5 (38.46%)	5 (29.41%)	15 (32.61%)
	Income = expenditure (Medium)	6 (37.50%)	5 (38.46%)	8 (47.06%)	19 (41.30%)
	Income < expenditure (low)	5 (32.25%)	3 (23.08%)	4 (23.53%)	12 (26.09%)
**Education Level**	Under diploma	5 (31.25%)	3 (23.08%)	3 (17.65%)	11 (23.91%)
	Diploma	4 (25.00%)	3 (23.08%)	4 (23.53%)	11 (23.91%)
	Undergraduate ^a^	3 (18.75%)	5 (38.46%)	7 (41.18%)	15 (32.61%)
	Graduate ^b^	4 (25.00%)	2 (15.38%)	3 (17.65%)	9 (19.57%)
**Employment Status**	Employed	8 (50.0%)	4 (30.77%)	10 (58.82%)	22 (45.83%)
	Non-Employed	8 (50.0%)	9 (69.23%)	7 (41.18%)	26 (54.17%)
Total Number of Respondents per City		16 (100%)	13 (100%)	17 (100%)	46 (100%)

Mean values are presented as Mean ± Standard Deviation (SD).

^a^Undergraduate refers to individuals with associate and bachelor’s degrees.

^b^Graduate indicates those with master’s or doctorate degrees.

**Fig 1:**
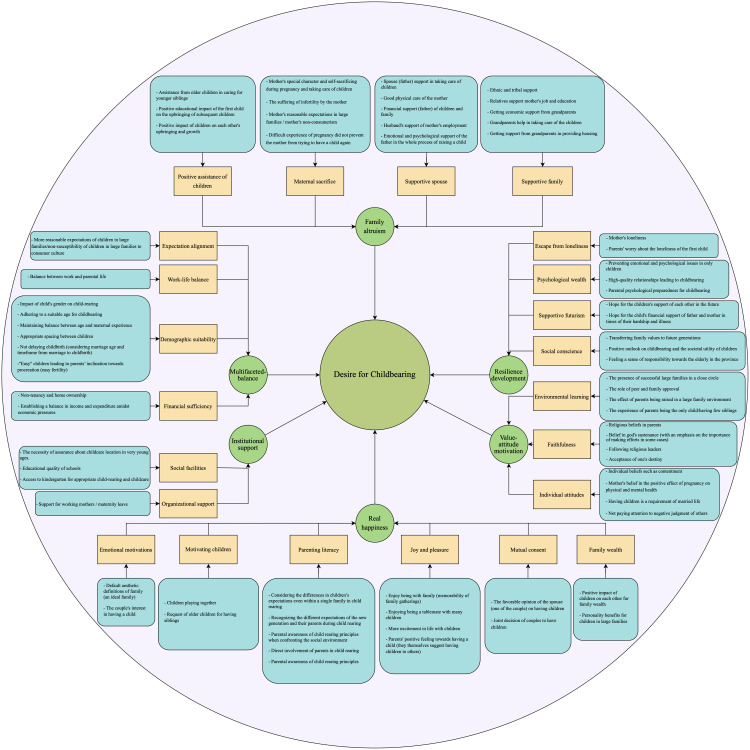
Hierarchical structure of themes, categories, and subcategories reflecting the perceptions of parents with two or more children who desire additional offspring.

Six overarching themes were identified through thematic analysis, each encompassing related categories and subcategories. These themes include: (1) Family Altruism, (2) Value–Attitude Motivation, (3) Real Happiness, (4) Resilience Development, (5) Institutional Support, and (6) Multifaceted Balance. A brief summary of each theme is provided below to support interpretation of participants’ motivations. Fig 1 illustrates the hierarchical structure of themes, categories, and subcategories, which is also summarized in S1 Table. S2 Table presents representative open codes for each subcategory.

1. Family Altruism: Participants described strong intergenerational and spousal support as key to making childrearing manageable and future childbearing desirable. Grandparents, spouses, and older siblings provided emotional, financial, and caregiving assistance, creating a cooperative family environment that reduced the burden of parenting.

*“Without the help of our families, we wouldn’t have even tried, because a child needs care, upbringing, and attention — and my mother really helped me a lot.”* (Participant reflections from Rasht (R1,6,7); Shiraz (S1,11,12); Mashhad (M1,7,8,9,12)).

2. Value–Attitude Motivation: Cultural upbringing, personal values, and religious beliefs shaped positive attitudes toward having more children. Many participants associated larger families with companionship, purpose, and spiritual fulfilment, reflecting internalized motivations beyond societal expectations.

*“I brought them into this world, I guide their upbringing, but ultimately, there’s a higher power protecting them.”* (Participant reflections from Rasht (R2,4); Shiraz (S6); Mashhad (M6,10,15)).

3. Real Happiness: Emotional gratification from parenting—including joy, identity, and family bonding—was a major motivator for continued childbearing. Participants found daily family life enriching and emphasized how children strengthened marital and sibling relationships.

*“My spouse grew up in a lively, bustling environment, with everyone gathering on weekends. That’s why they wanted to create the same atmosphere in our own family.”* (Participant reflections from Rasht (R7,8,12); Shiraz (S10,12); Mashhad (M7,9,10)).

4. Resilience Development: Childbearing was linked to enhanced emotional and social resilience. Parents viewed children as sources of companionship, psychological growth, future support, and civic contribution, suggesting that fertility decisions were grounded in both personal development and collective responsibility.

*“When families get older, they realize that if they’d had more children, they’d be better off now.”* (Participant reflections from Rasht (R4,13,14), Shiraz (S2,7)).

5. Institutional Support: Access to quality childcare, supportive workplaces, and equitable family policies significantly influenced childbearing decisions. Institutional resources enabled work-life balance, maternal employment, and shared parenting, mitigating structural barriers to family expansion.

*“She had 9 months of maternity leave, and those 9 months really helped us.”* (Participant reflections from Rasht (R9); Mashhad (M16)).

6. Multifaceted Balance: Participants evaluated emotional readiness, financial stability, and lifestyle compatibility before deciding on further childbearing. Successful balance was supported by shared caregiving, flexible employment, and values that emphasized cooperation and simplicity in family life.

*“I mostly felt that I became more understanding, more capable — like, behaviorally, I could connect better with my child. Back then, I was much younger and more immature. I think the best age to start having kids is from 20, because your mind is more ready for raising children.”* (Her first child was at age 14.) (Participant reflections from Rasht (R6,12); Shiraz (S8,9,10)).

## Discussion

This study examined the perspectives of Iranian parents with two or more children on planned childbearing. Six themes emerged: Family Altruism, Value–Attitude Motivation, Real Happiness, Resilience Development, Institutional Support, and Multifaceted Balance. These factors reflect emotional, cultural, psychological, and structural influences shaping fertility intentions.

Participants emphasized how support from extended family—especially spouses, grandparents, and older siblings—made parenting less burdensome and larger families more achievable. Grandparents provided childcare, housing, and financial help, echoing studies on intergenerational support reducing fertility barriers [38,39]. Spousal cooperation, particularly fathers’ emotional and financial involvement, fostered marital stability and reinforced shared parenting, consistent with literature linking male involvement to fertility desires [40,41]. Unlike childless individuals who cited fear and uncertainty [13], mothers here showed a readiness for sacrifice, suggesting maternal experience coupled with family support fosters resilience and receptivity to further childbearing. Older siblings’ caregiving further eased parenting demands, reinforcing household functioning and emotional development [42–45]. Recent research highlights how family structure and involvement, particularly among households with multiple children, naturally limits screen exposure in toddlers and encourages social interaction [46,47]. This aligns with our finding that intergenerational and sibling support systems enhance resilience and reduce reliance on passive caregiving tools like screens. These findings point to the importance of strengthening familial ecosystems in fertility-supportive strategies.

Planned childbearing was often grounded in childhood experiences, cultural values, and faith. Participants raised in large families desired similar environments, while those from smaller households recalled loneliness, emphasizing sibling companionship—findings aligned with the Theory of Planned Behavior and studies on social learning [13,14,48,49]. Faith-based beliefs, including trust in divine provision and religious authority, helped alleviate anxieties around financial or health concerns, consistent with research from Islamic and Western contexts [13,48,50]. Internally, many saw parenting as a source of contentment and purpose, despite challenges. Mothers in particular framed motherhood as identity-affirming, diverging from studies citing health concerns as deterrents among childless adults [13]. Some resisted societal judgment, showing self-directed values that nuance the TPB’s emphasis on normative pressure [51]. As our participants noted, personal values and beliefs significantly influence parenting choices. This resonates with ecological research showing that media outcomes depend heavily on contextual factors, including family values, media supervision, and socioeconomic environment [46,47]. Fertility interventions may be more effective when aligning with cultural values and personal meaning rather than relying solely on external incentives.

Parenting brought emotional fulfillment and family cohesion, motivating further childbearing. Participants highlighted the joy from everyday family interactions, marital enrichment, and sibling bonding—findings consistent with other qualitative studies [13,52]. Children were seen as enhancing spousal relationships and life satisfaction, contradicting literature emphasizing financial or social deterrents [52]. Mothers often described parenting as identity-building [53], and increased parenting literacy boosted confidence in family expansion. Rather than fearing modern challenges, participants expressed adaptive optimism, contrasting with studies that view societal pressures as barriers [52]. Spousal agreement also reinforced emotional foundations for family growth, echoing research linking joint decision-making to fertility desires [52,54]. Some described older children requesting siblings or offering care—an intra-family influence rarely captured in current literature. This dynamic supports developmental theories and highlights how real happiness from parenting reflects a lasting emotional investment rather than a compensatory desire [55,56]. Policies promoting emotional well-being and interpersonal satisfaction may reinforce fertility intentions.

Childbearing was viewed as enhancing emotional and social resilience through reduced loneliness, psychological enrichment, future security, and social contribution. Some cited emotional isolation in early marriage as prompting a desire for a more vibrant family life, aligning with studies on the psychological costs of singleton upbringing [14,57,58]. Parents valued sibling companionship for fostering emotional growth and long-term well-being, reflecting a generational shift toward emotional priorities in family planning [59,60]. Practical expectations were also evident; parents hoped siblings would care for each other and support aging parents—a motive consistent with findings on intergenerational reciprocity [61–63]. A few expressed civic motivations, seeing childbearing as a contribution to demographic sustainability and cultural continuity, echoing prior findings [13,52]. These insights suggest that fertility may be driven by both personal resilience and a future-oriented sense of duty, pointing to the need for policies that reinforce family solidarity and social cohesion.

External resources—especially quality childcare and supportive workplace policies—significantly shaped childbearing decisions. Accessible kindergartens and schools reduced parenting stress and supported children’s development, in line with studies linking early education infrastructure to fertility plans [64]. Reliable childcare enabled maternal employment and dual-earner households, reinforcing findings on the role of institutional support in gender equity and workforce participation [65]. Parents valued maternity leave and job flexibility, which facilitated bonding and caregiving without career compromise—consistent with other evidence [64]. Some reported greater paternal caregiving, reflecting cultural shifts that may complement policy initiatives. The need for institutional childcare options, as mentioned by participants, is further underscored by recent WHO and AAP guidelines on screen media. Where institutional support is lacking, screen time often replaces structured child-rearing, reinforcing the demand for policy interventions [46,47]. Thus, institutional support remains pivotal in reducing structural barriers and supporting family growth, especially when paired with evolving gender norms.

Participants weighed emotional readiness, financial security, and lifestyle coherence when considering further childbearing. Many emphasized that managing expectations, sustaining work-life balance, and ensuring material stability were central to these decisions, echoing earlier findings [66]. Some parents noted that children in larger families often adopt cooperative behaviors and modest expectations, easing pressures linked to consumer demands. Flexible work arrangements and shared caregiving were commonly used strategies to maintain parental presence, supporting Greenhaus and Beutell’s (1985) theory of inter-role conflict [67] and related studies on workplace flexibility and reproductive confidence [68]. Parents also valued close sibling bonds, often preferring minimal age gaps to encourage emotional closeness and social development. While some acknowledged risks of delayed childbearing [69], several noted that greater emotional maturity improved parenting quality, aligning with literature on optimal childbearing age [70,71]. Gender balance was valued, particularly in ethnic or tribal communities, though without strong gender bias [72]. Homeownership signaled stability, reinforcing findings that economic security and autonomy promote positive fertility intentions [73]. Policies that support financial stability, housing access, and flexible work options may help families realize their ideal size while maintaining emotional and economic balance.

## Conclusion

This study offers a nuanced understanding of planned childbearing among Iranian parents with two or more children, revealing that fertility intentions are shaped not solely by economic calculations but through a complex interplay of emotional, cultural, psychological, and structural factors. The six identified themes—*Family Altruism*, *Value–Attitude Motivation*, *Real Happiness*, *Resilience Development*, *Institutional Support*, and *Multifaceted Balance*—highlight the critical roles of intergenerational support, cultural identity, emotional fulfillment, adaptive resilience, and enabling environments in reproductive decision-making. These findings challenge deficit-based narratives about fertility decline by showing that many parents maintain a desire for family expansion when supported by meaningful relationships, personal values, and responsive institutions. To foster planned childbearing, policies must move beyond financial incentives and address the full spectrum of parental needs—strengthening family networks, aligning fertility interventions with cultural values, promoting emotional well-being, and ensuring access to flexible, equitable social infrastructure. Such a holistic approach can empower families to achieve their desired size while sustaining personal and societal well-being.

## Strengths and limitations

A key strength of this study is its focus on parents with two or more planned children—an underexplored group in prior research, which often emphasized fertility decline or childlessness. This perspective offers valuable insight into the positive drivers of continued childbearing in Iran. The study also benefits from purposeful sampling across three provinces—Rasht, Shiraz, and Mashhad—which were selected to capture a broad range of fertility rates and cultural, geographic, and socioeconomic diversity. While this enhances the representativeness of the findings, expanding sampling to additional provinces would further improve transferability across Iran’s full demographic spectrum. Additionally, future quantitative research is needed to assess the strength of associations and enhance the applicability of these qualitative findings. Socioeconomic status was assessed using a self-reported income–expenditure measure. In the Iranian context, direct income reporting is often unreliable due to sensitivity and low response rates. Therefore, we used a validated, culturally appropriate proxy measure alongside education and occupation data to improve accuracy, though it remains less precise than objective SES tools. Future studies may benefit from using more comprehensive socioeconomic indicators.

### Declaration of generative AI and AI-assisted technologies in the writing process

During the preparation of this manuscript, the authors used ChatGPT-4o to improve the structure and grammar of the text. All content was subsequently reviewed and edited by the authors, who take full responsibility for the final version of the manuscript.

## Supporting information

S1 Table Perception of parents who have two or more children and express a desire to have additional offspring. (DOCX)

S2 Table Perceptions of parents with two or more children regarding the desire for additional offspring: themes, categories, and representative open codes. (DOCX)
