# Supplementary material for: Desire for childbearing in the view of Iranian parents: A qualitative study
Source: PLoS One. 2025 Aug 22;20(8):e0330658. doi: 10.1371/journal.pone.0330658 (PMC12373215; doi:10.1371/journal.pone.0330658)
Supplement: S1 Table — (DOCX) [file pone.0330658.s001.docx]

| **S1 Table: Perception of parents who have two or more children and express a desire to have additional offspring.** | | |
| --- | --- | --- |
| **Themes** | **Category** | **Subcategory** |
| Family altruism | Supportive family | - Ethnic and tribal support - Relatives support mother's job and education - Getting economic support from grandparents - Grandparents help in taking care of the children - Getting support from grandparents in providing housing |
|  | *Supportive spouse* | - Spouse (father) support in taking care of children - Good physical care of the mother - Financial support (father) of children and family - Husband's support of mother's employment - Emotional and psychological support of the father in the whole process of raising a child |
|  | *Maternal sacrifice* | - Mother's special character and self-sacrificing during pregnancy and taking care of children - The suffering of infertility by the mother - Mother's reasonable expectations in large families / mother's non-consumerism - Difficult experience of pregnancy did not prevent the mother from trying to have a child again |
|  | *Positive assistance of children* | - Assistance from older children in caring for younger siblings - Positive educational impact of the first child on the upbringing of subsequent children - Positive impact of children on each other's upbringing and growth |
| Value-attitude motivation | *Environmental learning* | - The presence of successful large families in a close circle - The role of peer and family approval - The effect of parents being raised in a large family environment - The experience of parents being the only child/ having few siblings |
|  | *Faithfulness* | - Religious beliefs in parents - Belief in god's sustenance (with an emphasis on the importance of making efforts in some cases) - Following religious leaders - Acceptance of one's destiny |
|  | *Individual attitudes* | - Individual beliefs such as contentment - Mother's belief in the positive effect of pregnancy on physical and mental health - Having children is a requirement of married life - Not paying attention to negative judgment of others |
| Real happiness | *Parenting literacy* | - Considering the differences in children’s expectations even within a single family in child rearing - Recognizing the different expectations of the new generation and their parents during child rearing - Parental awareness of child rearing principles when confronting the social environment - Direct involvement of parents in child rearing - Parental awareness of child rearing principles |
|  | *Joy and pleasure* | - Enjoy being with family (memorability of family gatherings) - Enjoying being a tablemate with many children - More excitement in life with children - Parents' positive feeling towards having a child (they themselves suggest having children to others) |
|  | *Mutual consent* | - The favorable opinion of the spouse (one of the couple) on having children - Joint decision of couples to have children |
|  | *Motivating children* | - Children playing together - Request of older children for having siblings |
|  | *Emotional motivations* | - Default aesthetic definitions of family (an ideal family) - The couple's interest in having a child |
|  | *Family wealth* | - Positive impact of children on each other for family wealth - Personality benefits for children in large families |
| Resilience development | *Escape from loneliness* | - Mother's loneliness - Parents' worry about the loneliness of the first child |
|  | *Psychological wealth* | - Preventing emotional and psychological issues in only children - High-quality relationships leading to childbearing. - Parental psychological preparedness for childbearing |
|  | *Supportive futurism* | - Hope for the children's support of each other in the future. - Hope for the child's financial support of father and mother in times of their hardship and illness. |
|  | *Social conscience* | - Transferring family values to future generations - Positive outlook on childbearing and the societal utility of children - Feeling a sense of responsibility towards the elderly in the province |
| Institutional support | *Social facilities* | - The necessity of assurance about childcare location in very young ages. - Educational quality of schools - Access to kindergarten for appropriate child-rearing and childcare. |
|  | *Organizational support* | - Support for working mothers / maternity leave |
| Multifaceted- balance | *Expectation alignment* | - More reasonable expectations of children in large families/non-susceptibility of children in large families to consumer culture |
|  | *Work-life balance* | - Balance between work and parental life |
|  | *Demographic suitability* | - Impact of child's gender on child-rearing - Adhering to a suitable age for childbearing - Maintaining balance between age and maternal experience - Appropriate spacing between children - Not delaying childbirth (considering marriage age and timeframe from marriage to childbirth) - "easy" children leading to parents’ inclination towards procreation (easy fertility) |
|  | *Financial sufficiency* | - Non-tenancy and home ownership - Establishing a balance in income and expenditure amidst economic pressures |
