# Supplementary material for: Desire for childbearing in the view of Iranian parents: A qualitative study
Source: PLoS One. 2025 Aug 22;20(8):e0330658. doi: 10.1371/journal.pone.0330658 (PMC12373215; doi:10.1371/journal.pone.0330658)
Supplement: S2 Table — (DOCX) [file pone.0330658.s002.docx]

| **S2 Table. Perceptions of parents with two or more children regarding the desire for additional offspring: themes, categories, and representative open codes.** | | | |
| --- | --- | --- | --- |
| **Themes** | **Category** | **Subcategory** | **Representative Open Code** |
| Family altruism | Supportive family | Ethnic and tribal support | *“Even for naming the baby, like what name to choose, the elders gave us input.”* Rasht (R7), Shiraz (S1), Mashhad (M2,11) |
|  |  | Relatives support mother's job and education | *“My younger sister really loved children and I’m always grateful to her; she raised my son. She would take care of him so I could go to university and study. Getting help is really important. If it wasn’t for my sister, there would have been no one to watch my child, and I wouldn’t have been able to continue my studies or work.”* Shiraz (S1) |
|  |  | Getting economic support from grandparents | *“In the past, a couple could easily become stable and really didn’t need much help from parents. But nowadays, support is necessary, and the father of a young person has to help them.”* Rasht (R6,14,15); Shiraz (S9); Mashhad (M2,14) |
|  |  | Grandparents help in taking care of the children | *“Without the help of our families, we wouldn’t have even tried, because a child needs care, upbringing, and attention — and my mother really helped me a lot.”* Rasht (R1,6,7); Shiraz (S1,11,12); Mashhad (M1,7,8,9,12) |
|  |  | Getting support from grandparents in providing housing | *“At first, we lived with my parents.”* Rasht (R8,16); Shiraz (S1,2,9,13) |
|  | Supportive spouse | Spouse (father) support in taking care of children | *“Even my husband often took time off to take care of the kids, because in my job, I really can’t take much leave.”* Rasht (R1,2,6,7,10,14); Shiraz (S1,4,5); Mashhad (M2,8,15) |
|  |  | Good physical care of the mother | *“When I’m breastfeeding, my body feels very healthy and immune.”* Rasht (R9); Shiraz (S2,5) |
|  |  | Financial support (father) of children and family | *“Emotional maturity is like this: I got married, but I see it as my responsibility to work from morning to night, and that’s my way of showing respect and love for my wife and family.”* Rasht (R4,16) |
|  |  | Husband's support of mother's employment | *“Yes, he’s been really supportive — I even brought my husband here today because he’s been so helpful so I could at least be employed myself.”* Rasht (R9), Shiraz (S1), Mashhad (M6) |
|  |  | Emotional and psychological support of the father in the whole process of raising a child | *“It’s enough — really, my husband has helped me so much with the baby and the household. It’s not like I’ve been left alone; he’s never let me do the hard household tasks.”* Rasht (R4,7,9,15); Shiraz (S4,9); Mashhad (M6) |
|  | Maternal sacrifice | Mother's special character and self-sacrificing during pregnancy and taking care of children | *“Of course, nothing comes without challenges. I used to have lots of time for myself — classes, gym, going out with friends — but now most of my time goes to my children.”* Rasht (R2), Mashhad (M7,9,10,16) |
|  |  | The suffering of infertility by the mother | *“For some mothers, pregnancy and childbirth are hard; but once the child arrives and they experience its sweetness, they forget the hardships they had…”* Rasht (R3,4,8); Shiraz (S5,6,11); Mashhad (M1,7,8,11,13) |
|  |  | Mother's reasonable expectations in large families / mother's non-consumerism | *“Back then, we would search for and buy the cheapest clothes ourselves. These days, no one puts another child’s clothes on their own kid — but I did. I raised both my kids that way: the first child’s clothes were used for the second. Even when I went to the hospital for delivery, I had kept the newborn clothes and baby bag clean to use again. But nowadays, no one does that — no matter what, they insist on buying everything new. You can’t even do otherwise; people look at you in the hospital and mock you. Those women sitting and resting there, those new mothers — the ones who don’t have money, by God, they feel ashamed.”* Rasht (R4,13); Shiraz (S1,6,8); Mashhad (M7) |
|  |  | Difficult experience of pregnancy did not prevent the mother from trying to have a child again | *“Because my first delivery was a bit difficult, I was a little scared. That’s why I wanted some time between pregnancies, and I talked to the doctor, and they said it doesn’t have to be a natural delivery, and I had more experience now — all this led me to decide to have another child.”* Rasht (R10); Shiraz (S5); Mashhad (M4,7,12) |
|  | Positive assistance of children | Assistance from older children in caring for younger siblings | *“My oldest child has helped me a lot, especially with my fourth child. Even from a young age, they were very helpful.”* Rasht (R1,8) |
|  |  | Positive educational impact of the first child on the upbringing of subsequent children | *“The second and third children, in a way, see the first child as a role model. Even if we don’t directly talk to the second or third child, we can use the first and second to guide the younger ones. For example, in terms of schoolwork, the older ones are like their motivators, their examples. My second son is a bit more mischievous — not as obedient as the first — but no matter what, he has to study and behave well. He kind of listens to and imitates his older brother.”* Rasht (R8,10); Shiraz (S9,13) |
|  |  | Positive impact of children on each other's upbringing and growth | *“As the number of children increased, honestly, the parenting challenges became much less. For example, once you raise the first child, you barely need to spend time on the others—the first one ends up raising the rest.”* Rasht (R6,8,12,13), Shiraz (S1), Mashhad (M17) |
| Value-attitude motivation | Environmental learning | The presence of successful large families in a close circle | *“Before this, we went to a ceremony for some local representatives, and we saw a family — believe me, for the first time I felt envious — five or six brothers, all wearing suits, standing at the entrance, showing each other so much respect. We were four brothers and two sisters, and we also had special respect for each other, but when I saw them, so well-dressed, so respectful, I really liked it. Experiences like that contributed to us deciding to have three children.”* Rasht (R7,10); Mashhad (M1,8,11,16) |
|  |  | The role of peer and family approval | *“My husband’s family really insisted that we have children sooner; they wanted to have a grandchild since their family was small. My own family didn’t mind either and told us to have kids. Their advice definitely had an influence.”* Rasht (R2,6,10,14,15), Shiraz (S2,5,8,11,13), Mashhad (M7,8,9,15) |
|  |  | The effect of parents being raised in a large family environment | *“Overall, I myself enjoyed interacting with big families. I even see my own parents doting on their grandchildren, taking them out, and it rekindles that sense of youth in them, reminding them of when we were children.”* Rasht (R2,4,7,9,10); Shiraz (S1,2,6,8); Mashhad (M2,4,14) |
|  |  | The experience of parents being the only child/ having few siblings | *“One reason is that my husband is an only child himself, and he grew up alone.”* Mashhad (M17) |
|  | Faithfulness | Religious beliefs in parents | *“For the first two years, we didn’t plan to have kids. After two years, we visited Imam Reza’s shrine, did an istikhara (prayer for guidance), and it came out positive, so we decided to have children.”* Rasht (R4,5,9,12); Shiraz (S6,7,8,12); Mashhad (M2,3,14) |
|  |  | Belief in god's sustenance (with an emphasis on the importance of making efforts in some cases) | - *“Any child that God brings into this world, He also provides for. You shouldn’t worry so much about not having a house or car.”* - *“Every child comes with their own blessings.”* Rasht (R2,4,7,13); Shiraz (S6,10,12); Mashhad (M4,7,13) |
|  |  | Following religious leaders | *“As the religious leader has said that having more children is good, we decided to follow his guidance and have another child.”* Rasht (R12); Shiraz (S6); Mashhad (M10,11) |
|  |  | Acceptance of one's destiny | - *“I’m not worried about my children; God watches over them.”* - *“I brought them into this world, I guide their upbringing, but ultimately, there’s a higher power protecting them.”* Rasht (R2,4); Shiraz (S6); Mashhad (M6,10,15) |
|  | Individual attitudes | Individual beliefs such as contentment | *“I feel satisfied about having children. Overall, I enjoy having children.”* Shiraz (S10); Mashhad (M4,12) |
|  |  | Mother's belief in the positive effect of pregnancy on physical and mental health | *“When I breastfeed, my body feels very healthy and strong.”* Rasht (R9); Shiraz (S2,5) |
|  |  | Having children is a requirement of married life | *“To be honest, like other couples, children are a necessary part of married life. After marriage, everyone tries to have kids.”* Mashhad (M1,12) |
|  |  | Not paying attention to negative judgment of others | *“My family used to say, ‘Having many kids isn’t good…’ they were the obstacle, but we were enthusiastic.”* Rasht (R10); Shiraz (S6) |
| Real happiness | Parenting literacy | Considering the differences in children’s expectations even within a single family in child rearing | *“A lot of problems come when the kids get older — school issues, homework, and all that. If I had a third child now, most of my time would go to that baby, but at the same time, my daughter is just starting first grade and I need to work with her too.”* Rasht (R1,10,12,15,16); Shiraz (S10,11); Mashhad (M1,2,17) |
|  |  | Recognizing the different expectations of the new generation and their parents during child rearing | *“The youngest child has more talent than the first child. My youngest (second grader) downloads and installs computer games, but my first child didn’t have those resources and couldn’t do it. He only learned after middle school. This makes the youngest child smarter and more knowledgeable.”* Rasht (R2,4,5,7,8,13,14); Shiraz (S5,9,12,13); Mashhad (M5,7,16) |
|  |  | Parental awareness of child rearing principles when confronting the social environment | *“This concern isn’t just mine — it’s something every parent worries about: how will the child grow up, who will they hang out with, who will come into their life? My first child was a boy, and little boys are so playful. I was very strict and sensitive about his choice of friends — worried about bad influences.”* Rasht (R5,7,8,12,14); Shiraz (S3,6,7,10,12); Mashhad (M2) |
|  |  | Direct involvement of parents in child rearing | - *“If parents are present, they can have more and better children. That is, if the father and mother work eight hours and then are physically present at home for eight hours, they can raise healthy kids. There’s a meaningful relationship between the lack of physical presence or face-to-face interaction between parents and children at home and the likelihood of children being exposed to social harm later.”* - *“Parental presence, especially during ages 14–15, is very important.”* Rasht (R15); Mashhad (M16) |
|  |  | Parental awareness of child rearing principles | *“We went to a therapist, and the therapist said that ideally, you should have gone through therapy even before having kids to learn the principles of child-rearing. Every parent has their own ethical framework — some parts can be corrected, and some behaviors we teach, so that the child won’t be harmed in the future.”* Rasht (R2,6,9,12,15); Shiraz (S6,10,12); Mashhad (M2,8,15,16) |
|  | Joy and pleasure | Enjoy being with family (memorability of family gatherings) | *“My spouse grew up in a lively, bustling environment, with everyone gathering on weekends. That’s why they wanted to create the same atmosphere in our own family.”* Rasht (R7,8,12); Shiraz (S10,12); Mashhad (M7,9,10) |
|  |  | Enjoying being a tablemate with many children | *“I think having three kids is best. When we sit at the table together, I enjoy it. It’s like when you sit with four friends at a table — it’s much more fun than with just two people.”* Rasht (R4,7) |
|  |  | More excitement in life with children | *“When our second child was born, the excitement in our life increased a lot. Now, both my spouse and I want to have another child to make our life even more beautiful.”* Rasht (R2,4); Shiraz (S1,6); Mashhad (M5,7) |
|  |  | Parents' positive feeling towards having a child (they themselves suggest having children to others) | *“Whenever I meet someone, I tell them one or two kids isn’t enough — children should have siblings to be friends with.”* Rasht (R4,6); Shiraz (S4,6,13); Mashhad (M1) |
|  | Mutual consent | The favorable opinion of the spouse (one of the couple) on having children | *“My husband really wanted us to have at least one child, and now he even says, if we had the means, it wouldn’t have been bad — if you had wanted another, we could’ve had one more.”* Rasht (R1,12,13); Shiraz (S2,4,13); Mashhad (M1,8,10,14,17) |
|  |  | Joint decision of couples to have children | *“You can’t just live based only on your own ideas; both partners should know each other first and then start a family, because if a couple has a child but ends up heading toward divorce, that’s no longer a real family.”* Rasht (R10), Shiraz (S1,6,8) |
|  | Motivating children | Children playing together | *“My children, since they are several, play together, but the parents of only children often ask us to let their child come over and play with ours for a few hours.”* Rasht (R8,9); Mashhad (M1) |
|  |  | Request of older children for having siblings | - *“My first child, a boy, when he went to school, would say, ‘My friends have brothers and sisters, but I’m alone.’ Mainly because of his loneliness, we had another child.”* - *“He was always shy and isolated. Even when we went out, he would say, ‘Lucky for my cousin, he has a brother or sister, but I don’t.’ Mostly, we did it for him.”* Rasht (R6,8,9); Shiraz (S2,7); Mashhad (M14) |
|  | Emotional motivations | Default aesthetic definitions of family (an ideal family) | - *“An empty house feels lifeless.”* - *“A beautiful family is one that has many children.”* Rasht (R2,3,5,10,12,13); Shiraz (S2,3,6,8); Mashhad (M5,6,10) |
|  |  | The couple's interest in having a child | \| *“We really loved children. Honestly, having a child brings such joy — it’s hard to put into words. Yes, there are many economic problems, but when you come home and the child runs into your arms, it’s a whole different feeling.”* Rasht (R4,6,7); Shiraz (S3,9,11); Mashhad (M4,12,17) \| \| --- \| |
|  | Family wealth | Positive impact of children on each other for family wealth | *“The positive influences that children have on one another contribute to the elevation and enrichment of the family. Children affect each other more than we, as parents, influence them.”* Shiraz (S1) |
|  |  | Personality benefits for children in large families | *“Well, when I gave birth to my second child, I saw a huge change in my first child’s behavior — before, she would play alone or cry all the time at parties because she felt lonely, but after her brother was born, things got so much better.”* Rasht (R6,16); Mashhad (M16,17) |
| Resilience development | Escape from loneliness | Mother's loneliness | *“Well, when I got married, my husband worked from morning to night, and I only saw him at night. My house was also very far from my mother’s house, and I felt an intense loneliness.”* Mashhad (M17) |
|  |  | Parents' worry about the loneliness of the first child | *“In my opinion, one of the reasons kids become depressed or isolated or turn to social media is loneliness. That’s one of the reasons.”* Rasht (R8,9); Shiraz (S5,7); Mashhad (M16) |
|  | Psychological wealth | Preventing emotional and psychological issues in only children | *“For example, my cousin’s daughter is an only child, and she’s really unhappy and dissatisfied, even though her financial situation is excellent. But she’s still thinking about having another child. She’s about one or two years older than me, and her child is about one or two years younger than mine. She married later, and even now, she’s still thinking, ‘I wish I had another child, a sibling for my kid—like a brother or sister.’”* Rasht (R2,6,8,14), Shiraz (S2,6,11,12), Mashhad (M4,8,11,17) |
|  |  | High-quality relationships leading to childbearing | *“I felt so much happiness and peace next to my husband that I thought: why not share more things with this person?”* Shiraz (S2), Mashhad (M7) |
|  |  | Parental psychological preparedness for childbearing | *“Both the wife and husband should be ready and come to the decision together to have a child.”* Rasht (R3); Shiraz (S8); Mashhad (M2,3,15,17) |
|  | Supportive futurism | Hope for the children's support of each other in the future | - *“This togetherness and unity that forms between siblings.”* - *“The fact that siblings are close and united gives me peace of mind. Even if I’m gone one day, they will have each other’s back.”* Rasht (R2,8); Shiraz (S4,13); Mashhad (M1,2,3) |
|  |  | Hope for the child's financial support of father and mother in times of their hardship and illness | - *“When families get older, they realize that if they’d had more children, they’d be better off now.”* - *“In old age, there’s no one to help them. The motivation for having kids in past decades was so the children could help out on the farm, help with expenses, be their ‘cane in old age.’ (Now it’s changed.)”* Rasht (R4,13,14), Shiraz (S2,7) |
|  | Social conscience | Transferring family values to future generations | - *“I personally really love children, and there’s also this feeling that you want your lineage to continue.”* - *“I wanted to give my children the love that I had received.”* Rasht (R4), Mashhad (M14) |
|  |  | Positive outlook on childbearing and the societal utility of children | *“Gradually, this child became a goal for me and my husband — to raise them to achieve something and be useful for the future.”* Rasht (R4,9,13); Shiraz (S8,11); Mashhad (M5,10) |
|  |  | Feeling a sense of responsibility towards the elderly in the province | - *“They say the population is aging, moving toward old age, so you need to have kids.”* - *“The country will shut down if there are no young people.”* Rasht (R6); Shiraz (S6); Mashhad (M1) |
| Institutional support | Social facilities | The necessity of assurance about childcare location in very young ages | *“Especially when the kids are very young, you have to take a few months off to stay home with them until they reach an age where you can leave them with someone. Our own preference has always been that they not go to daycare or anything like that but stay within the family environment.”* Shiraz (S1,12) |
|  |  | Educational quality of schools | - Are his grades good? *“Yes, thank God, his grades are good.”* - Do you help him yourself? *“No, it was the school he went to. Everything stayed at school — we didn’t need to do anything at home. Now, with the younger one who’s about to start school, it’s much harder in public schools. In the previous school, he wouldn’t even bring home a pencil — they handled everything at school. Hopefully, if we can manage, we’ll send him to private school again.”* - Were you more satisfied with the private school? *“Very much. It was bilingual, they worked with them in English, they had weekly celebrations and fun activities every Wednesday. The school was excellent. Now they’re missing out on all that...”* Shiraz (S11) |
|  |  | Access to kindergarten for appropriate child-rearing and childcare | *“I mostly took him to the park, and after age three, I enrolled him in kindergarten so he could play with other kids. I took him to kindergarten, to the park… I didn’t let him just stay in the alley.”* Rasht (R8); Shiraz (S2) |
|  | Organizational support | Support for working mothers / maternity leave | *“She had 9 months of maternity leave, and those 9 months really helped us.”* Rasht (R9); Mashhad (M16) |
| Multifaceted- balance | Expectation alignment | More reasonable expectations of children in large families/non-susceptibility of children in large families to consumer culture | *“I lived in a way that taught my kids: if I have money, I will buy something myself — they don’t need to ask or push. Their expectations are not high; they know I will get it if I can, without them demanding or crying for it. We, as parents, need to raise our kids to be like us. My kids are patient, they’re content. We raised them so their expectations wouldn’t be high — they don’t go around saying, ‘other kids have this, so I should have it too.’ They’re not caught up in that mindset at all.”* Rasht (R8,9,12,16); Shiraz (S5,6,12) |
|  | Work-life balance | Balance between work and parental life | *“That has a big effect on the child’s behavior and manners. For example, my sister, who was a working woman and a faculty member, enrolled her child in kindergarten from six months old. In some ways, that’s good, and in some ways, it’s not, because the child grows up very rule-bound from the start, which is good in some respects.”* Rasht (R15); Shiraz (S2,8,9); Mashhad (M16,17) |
|  | Demographic suitability | Impact of child's gender on child-rearing | *“Actually, I prefer daughters. When I was pregnant with my third child, I prayed, ‘God, I’m not ungrateful, but I really don’t want my daughter to be an only child.’ Because when daughters have sisters, later in life when the parents are gone, they have a companion, someone by their side. Boys usually just go their own way. Sure, boys also like having a brother, but in my opinion, girls need a sister more.”* Rasht (R2,5,6,9,10,11), Shiraz (S1,5,13), Mashhad (M3,2,5,17) |
|  |  | Adhering to a suitable age for childbearing | - *“I had my first child about two years after marriage (at age 18).”* - *“In fact, whenever someone marries young, I tell them: don’t rush, but after at least a year or two of marriage, go ahead and start having kids.”* Rasht (R1,8,10,15), Shiraz (S1,5,8,10) |
|  |  | Maintaining balance between age and maternal experience | *“I mostly felt that I became more understanding, more capable — like, behaviorally, I could connect better with my child. Back then, I was much younger and more immature. I think the best age to start having kids is from 20, because your mind is more ready for raising children.”* (Her first child was at age 14.) Rasht (R6,12); Shiraz (S8,9,10) |
|  |  | Appropriate spacing between children | *“My sisters and I had a big age gap, so we weren’t that close growing up. But my husband had lots of playmates when he was little because his siblings were close in age. So, I wanted my own kids to be close in age too, so they’d be closer to each other.”* Rasht (R8,9,10,12,15); Shiraz (S1,3,6,7,10,12); Mashhad (M1,2,7,8) |
|  |  | Not delaying childbirth (considering marriage age and timeframe from marriage to childbirth) | - *“We married a long time ago, around 2000. Back then, it wasn’t like today when people wait two or three years. I got pregnant just two or three months after marriage.”* - *“Yes, I wanted to have a child soon and feel what it’s like to be a mother... I really just wanted that sense of motherhood.”* Rasht (R2,5,7,9), Shiraz (S1,3,10,11,12) |
|  |  | "Easy" children leading to parents’ inclination towards procreation (easy fertility) | *“My son was so quiet and calm; you wouldn’t even hear a sound. Usually little boys are wild, climbing everywhere, but he was nothing like that, even now. For example, if he’s hungry, he won’t say anything; you have to ask him if he wants food. But my third daughter has really given me a hard time.”* Rasht (R5,7); Shiraz (S2,9); Mashhad (M7) |
|  | Financial sufficiency | Non-tenancy and home ownership | *“Because I wasn’t a renter myself, I don’t know what renters go through. But they say renters are always on the move, and it’s hard for them to relocate and move their stuff. If they have three kids, their belongings are tripled—who’s going to move all that from one house to another? If you notice, most people who are renting have only one child—unless they’re well-off and own a home, then they can continue having more.”* Rasht (R5,7,8,12,13), Shiraz (S3,6,7,11), Mashhad (M11) |
|  |  | Establishing a balance in income and expenditure amidst economic pressures | *“These days, look how much rent has gone up. You need to put down 600 million [rial] just for the deposit, plus rent. And with the salaries they pay us, by the end of the month, we get our paycheck, and three million goes to rent, to loans, and we’re left with nothing.”* Shiraz (S2,5), Mashhad (M14) |
| **Note**: One or two representative open codes are shown per subcategory; all participant codes listed expressed similar views. | | | |
